# Memorisation and implicit perceptual learning are enhanced for preferred musical intervals and chords

**DOI:** 10.3758/s13423-021-01922-z

**Published:** 2021-05-04

**Authors:** Pietro Sarasso, Pasqualina Perna, Paolo Barbieri, Marco Neppi-Modona, Katiuscia Sacco, Irene Ronga

**Affiliations:** BIP (BraIn Plasticity and behaviour changes) Research Group, Department of Psychology, University of Turin, Via Verdi 10, 10124 Turin, Italy

**Keywords:** Neuroaesthetics, Perceptual learning, Memory, Attention, Sound recognition

## Abstract

Is it true that we learn better what we like? Current neuroaesthetic and neurocomputational models of aesthetic appreciation postulate the existence of a correlation between aesthetic appreciation and learning. However, even though aesthetic appreciation has been associated with attentional enhancements, systematic evidence demonstrating its influence on learning processes is still lacking. Here, in two experiments, we investigated the relationship between aesthetic preferences for consonance versus dissonance and the memorisation of musical intervals and chords. In Experiment 1, 60 participants were first asked to memorise and evaluate arpeggiated triad chords (*memorisation phase*), then, following a distraction task, chords’ memorisation accuracy was measured (*recognition phase*). Memorisation resulted to be significantly enhanced for subjectively preferred as compared with non-preferred chords. To explore the possible neural mechanisms underlying these results, we performed an EEG study, directed to investigate implicit perceptual learning dynamics (Experiment 2). Through an auditory mismatch detection paradigm, electrophysiological responses to standard/deviant intervals were recorded, while participants were asked to evaluate the beauty of the intervals. We found a significant trial-by-trial correlation between subjective aesthetic judgements and single trial amplitude fluctuations of the ERP attention-related N1 component. Moreover, implicit perceptual learning, expressed by larger mismatch detection responses, was enhanced for more appreciated intervals. Altogether, our results showed the existence of a relationship between aesthetic appreciation and implicit learning dynamics as well as higher-order learning processes, such as memorisation. This finding might suggest possible future applications in different research domains such as teaching and rehabilitation of memory and attentional deficits.

## Introduction

In the present study, we propose a preliminary investigation of the relationship between perceptual learning and subjective aesthetic appreciation. It is with Baumgarten (1750) that the study of the nature of beauty gained its actual name (*epistêmê aisthetikê*, i.e. *aesthetics*, the science of what is sensed), reflecting its original function as an alternative approach to the philosophy of knowledge (Gross, 2002); in Baumgarten’s words: “the science of sensory knowledge directed toward beauty” (Berleant, 2015). More recently, following Dewey’s holistic approach (Stroud, 2010; Wong, 2007), the relationship between learning\knowledge-acquisition and aesthetic appreciation has extensively been investigated in teaching research (Uhrmacher, 2009) and progressively redefined through neurocomputational and psychological modeling (Muth & Carbon, 2013; Perlovsky & Schoeller, 2019; Sarasso, Neppi-Modona, Sacco & Ronga, 2020a; Sarasso, Ronga Neppi-Modona & Sacco 2021b; Schmidhuber, 2009; Schoeller, 2015; Schoeller & Perlovsky, 2016; Van de Cruys & Wagemans, 2011). Furthermore, neuroimaging studies found enhanced sensory activations during the perception of objects valued as beautiful as opposed to less appreciated ones (Kirsch, Urgesi & Cross, 2016; Nadal, 2013). This hyperactivation might subtend a learning-oriented attentional modulation (Kirsch et al., 2016; Nadal, 2013; Sarasso et al., 2020a) finalized to maintain the attentional focus on beautiful objects’ perceptual features (the so-called aesthetic attitude; Kingstone, Miller, Chatterjee & Vartanian, 2016; Gallese & Guerra, 2012; Stolnitz, 1978) and might represent a signal triggering aesthetic appreciation (Vartanian & Goel, 2004). Nevertheless, the link between implicit and explicit learning and aesthetic appreciation still lacks systematic empirical evidence.

Here, we investigated the relation between liking and learning in the domain of harmonic preferences. Consonant musical intervals and chords, while sharing similar single-note frequencies with dissonant ones, are known to induce greater aesthetic appreciation (Bowling & Purves, 2015; Bowling et al., 2017) and attentional engagement, most likely because interpreted by the nervous system as informationally more profitable in terms of signal-to-noise ratio (Sarasso, et al., 2020a). However, consonance, attentional advantage, and preference do not automatically correlate. In addition to such biologically hard-wired generalist trends, individual differences in aesthetic preference (especially when comparing consonant and mildly dissonant intervals and chords) are often observed (Lahdelma & Eerola, 2016a; McDermott et al., 2010; McDermott et al., 2016; Plantinga & Trehub, 2014). Individual fluctuations in preference for consonance/dissonance might be greatly influenced by expertise and previous listening experience (Lahdelma & Eerola, 2020). Moreover, beside psychoacoustic features, individual musical experience (i.e. musical sophistication) and enculturation (i.e. cultural familiarity; Lahdelma & Eerola, 2020) might play an important role in determining preference for consonance/dissonance (Lahdelma & Eerola, 2016a, 2016b). For example, the consonant major triad is more common than the mildly dissonant diminished triad in Western music. Therefore, following mere exposure (Zajonc, 2001), the consonant triad is expected to sound generally more attractive by participants who are acquainted with common-practice music. As a further example, tritone chords were forbidden in medieval times and defined as the *diabulus in musica* (*the devil in music*), and are generally avoided in Western harmony. Not for nothing, the tritone is commonly not a very preferred sonority in Western culture, even if it is not the objectively most dissonant chord (Lahdelma & Eerola, 2016a). In other words, when chords are culturally familiar, subjective preferences might be related to specific cultural factors rather than to purely acoustic factors; thus, the correlation between subjective preference and consonance is not as high as when the stimuli are unfamiliar (Lahdelma & Eerola, 2020).

To explore the relation between learning dynamics and subjective aesthetic judgements, we designed two different experimental paradigms employing more and less consonant chords (Experiment 1) and intervals (Experiment 2). Experiment 1, a behavioural memorisation-recognition task, is directed to investigate higher-order learning mechanisms, such as memorisation. Experiment 2, an EEG mismatch detection paradigm, is aimed at measuring the electrophysiological indexes of (lower-level) implicit perceptual learning mechanisms.

In Experiment 1, during the memorisation phase, participants listened to a series of either major or diminished arpeggiated triads (i.e. a sequence of three notes) and were asked to evaluate their beauty. During the successive recognition phase, the same arpeggiated triad chords were also intermixed with others that were not previously presented. Participants judged whether they had previously heard each triad. If, as hypothesized, learning phenomena are correlated with aesthetic appreciation, we expect to find increased memorisation accuracy for more appreciated chords, irrespective of their specific category (i.e. major vs. diminished). Conversely, if learning is dependent on a specific stimulus category (e.g., memorisation is better for major triad chords as compared with diminished ones), we should conclude that memorisation is specifically driven by some specific stimulus features, rather that subjective aesthetic judgements.

In Experiment 2, we intended to explore the neural mechanisms underlying a possible relation between subjective aesthetic judgements (AJs) and learning dynamics. To this aim, we exploited a modified version of a classical EEG mismatch detection paradigm (i.e. oddball tasks), specifically designed to collect trial-by-trial AJs. Oddball paradigms are usually employed to study electrophysiological mismatch detection responses associated with the identification of unexpected events such as infrequent sounds (Halgren et al., 1980; Näätänen et al., 2007). The presentation of an oddball (rare) sound, embedded in a stream of standard sounds, induces an enhancement of auditory evoked responses that can be recorded with the EEG (Justen & Herbert, 2018; Kennan et al., 2002; Sams et al., 1985). Mismatch detection responses are considered a well-validated index of implicit perceptual learning of sensory regularities (Garrido et al., 2009; Garrido et al., 2016; Rose et al., 2005). We registered EEG responses to standard and deviant more and less consonant musical just intervals (i.e. two simultaneously presented notes), while participants rated the beauty of each presented interval. Through this experiment, we aimed to assess the presence of a positive correlation between subjective AJs and successful learning of sensory regularities. This result would be twofold: (1) replication of previous findings indicating that more appreciated sounds induce an automatic attentional capture, expressed by the enhancement of attention-related components of the auditory evoked response such as N1 (Sarasso, Ronga, et al., 2019); (2) in accordance with previous studies investigating mismatch detection responses to occasional deviant stimuli (Näätänen et al., 2007; Wiens et al., 2019), more negative voltages should be recorded following the presentation of deviant as compared with standard intervals, within the latency range corresponding to mismatch detection responses (Justen & Herbert, 2018; Sams et al., 1985). More importantly for the present study, in line with the hypothesized link between implicit perceptual learning and beauty experience, we expect to observe greater mismatch detection responses for more appreciated intervals (indicating more effective perceptual learning dynamics).

A negative result (i.e. the absence of a correlation between subjective AJs, the enhancement of attentional-related EEG components, and the amplification of mismatch detection responses) would instead challenge our hypothesis suggesting a direct link between aesthetic appreciation and successful learning dynamics.

## Experiment 1: Memorisation task

### Methods

#### Participants

Sixty healthy young volunteers participated in the study (32 females; age: 23.3±2.02 years; education: 15.23±1.57 years). We excluded volunteers with a formal musical training as well as professional players and singers. All participants gave their written informed consent to participate in the study. The study conformed to the standards required by the Declaration of Helsinki and was approved by the local ethics committee (University of Turin, protocol number 121724). Sample size (*N* = 60) was a priori determined through a power analysis based on the effect size obtained in a pilot experiment identical to the main experiment, involving 14 additional participants and exploring memorisation accuracies (ACC) between preferred and non-preferred three-note arpeggiated chords (see Data Analysis section; Cohen’s *d* = 0.474, α = 0.05, required power = 0.95). Moreover, the sample size closely matched that of a previous study addressing the effect of aesthetic judgements on perceptual performances (Sarasso et al., 2020b).

#### Apparatus

Participants sat at a table in a fixed position, distant 60 cm from a loudspeaker and from a 53 cm (diagonal) computer screen, with the screen centre and the loudspeaker aligned with the participants’ trunk midline. The subjects’ left arm was resting on the corresponding leg, while the right hand was placed over the computer keyboard placed on the desk, ready to respond.

#### Stimuli

Chords were created with Csound software (https://csound.com/), which allowed to specify the frequency (Hz) of single notes composing the arpeggiated triad chords. Coherently with previous studies (Sarasso, Ronga, et al., 2019b), to exclude potential confounding effects, we chose to avoid any recognisable instrument timbre. Synthetic sounds in Experiments 1 and 2 were “played” by Csound “virtual” instrument vco2, which implemented a band-limited oscillator. As in previous studies (Rogers & Levitin, 2007), we created chords according to just intonation (or pure intonation). Just intonation is the tuning of musical chords or intervals as whole number ratios (such as 4:5:6 for major chords; 3:2 for fifths, or 45:32 for tritone intervals) between the frequencies of the single notes composing the chord (pitches presented sequentially in the current study), or interval (pitches presented simultaneously in the current study). Although equal temperament, rather than just intonation, is the most common tuning procedure in consonance/dissonance studies (Lahdelma & Eerola, 2020), we opted for just intonation because it more closely replicated the procedures of previous studies investigating learning and memory for consonant versus dissonant sounds (Rogers & Levitin, 2007) and electrophysiological correlates of harmonic preferences (Sarasso, Ronga, et al., 2019b).

Single notes were played sequentially with a duration of 0.5 s, with a 0.5 s silent interval between notes, for a total duration of 2.5 s per chord. Chord types (major and diminished) were defined by the different ratio between the frequency of the three notes composing the chord. Consonance also depends on the same ratio: the smaller the numbers that define the ratio, the more consonant will be the resulting chord (Plomp & Levelt, 1965). Major (more consonant) triad chords were composed by notes with a frequency ratio of 4:5:6; diminished (less consonant) chords had a frequency ratio of 160:192:231. We created four chords per type by varying the frequency of the lowest (Hz) note (280, 290, 300, and 310 Hz) and the order of presentation of the three single notes. The ordering of the pitches of single notes composing the arpeggiated triads was not constant (i.e. from low to high or vice versa), but changed, so that the task actually required to memorise relations between pitches rather than simply recognising the pitch of the root note. Table 1 reports the frequency of the three notes employed in the experiment. All chords were played via a loudspeaker at the output intensity of 65 dB delivered through E-Prime presentation software (Psychology Software Tools, Inc. USA).

**Table 1:** Frequency of the three notes employed in the experiment

Chord name	Frequency of note1 (Hz)	Frequency of note2 (Hz)	Frequency of note3 (Hz)
MAJ 1	310	465	387.5
MAJ 2	362.5	290	435
MAJ 3	350	420	280
MAJ 4	450	300	375
DIM 1	310	447.5	372
DIM 2	348	290	418.7
DIM 3	336	404.2	280
DIM 4	433.1	300	360
Run number	Chords presented in the memorisation phase	Chords presented in the recall phase	
1	MAJ 1; MAJ 2; DIM 3; DIM 4	MAJ 1; MAJ 2; MAJ3; MAJ4; DIM1; DIM2; DIM 3; DIM 4	
2	MAJ 2; MAJ 3; DIM 1; DIM 4	MAJ 1; MAJ 2; MAJ3; MAJ4; DIM1; DIM2; DIM 3; DIM 4	
3	MAJ 1; MAJ 4; DIM 2; DIM 3	MAJ 1; MAJ 2; MAJ3; MAJ4; DIM1; DIM2; DIM 3; DIM 4	
4	MAJ 3; MAJ 4; DIM 1; DIM 2	MAJ 1; MAJ 2; MAJ3; MAJ4; DIM1; DIM2; DIM 3; DIM 4	

*Note.* Top panel: The frequency (Hz) of the 0.5 s single notes composing the arpeggiated triad chords. MAJ and DIM chords were defined by a 4:5:6 and a 160:192:231 ratio between the frequency of single notes (note1; note2; note3), respectively. The ordering of the pitches in the arpeggiated triads systematically varied so that participants were actually required to memorise the relations between pitches. Bottom panel: The 4 chords presented in the memorisation and recall phases of in each run. Runs were numbered from 1 to 4 for display reasons only: participants performed the 4 runs in a randomized order. MAJ = Major triad chord; DIM = Diminished triad chord.

#### Procedure

**Fig. 1:**
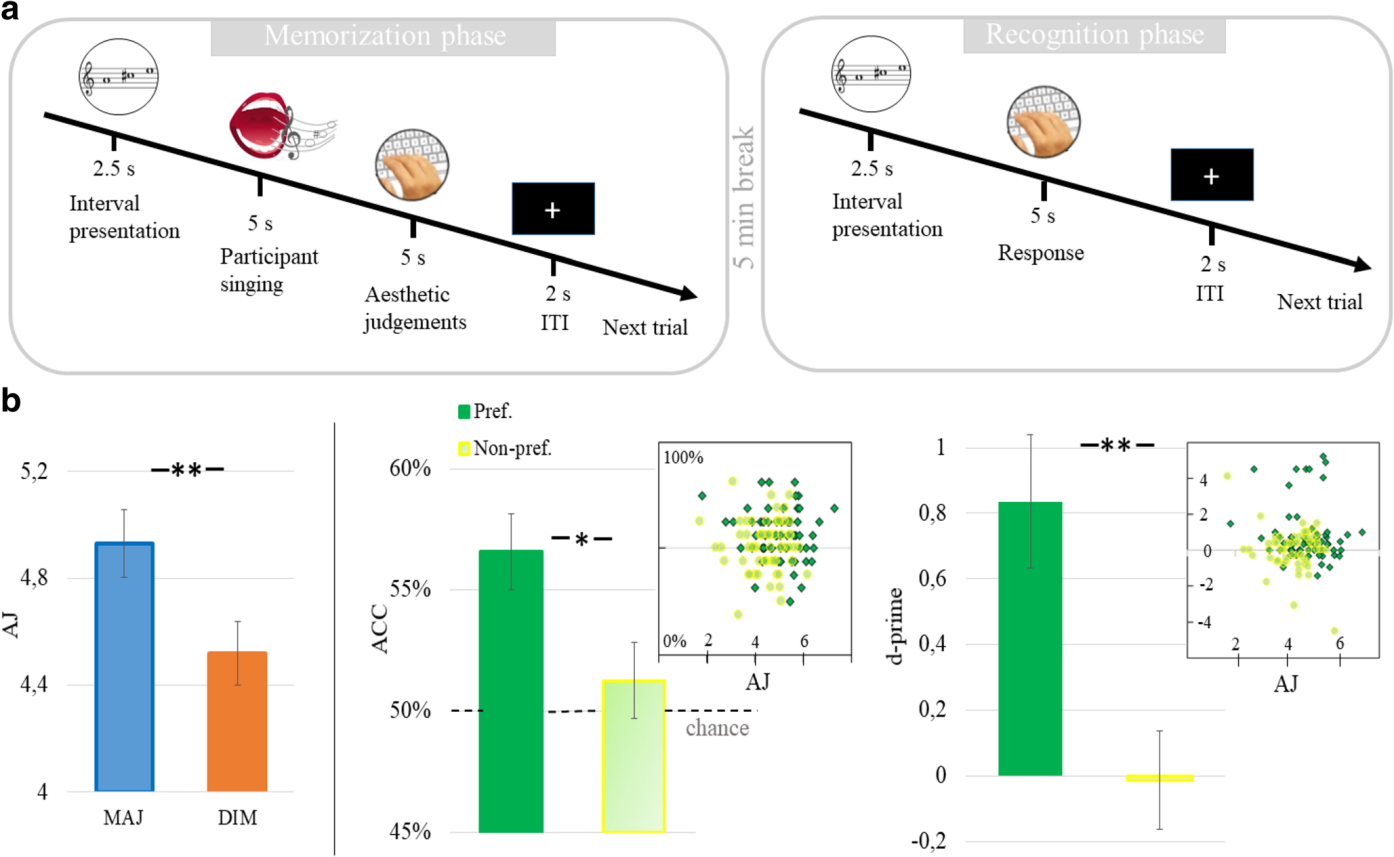
**a** **Experimental procedures.** In each of the four runs participants performed a memorisation and a recognition task. **b Behavioural results.** The bottom-left panel displays aesthetic judgements corresponding to MAJ, DIM chords. The right bottom panel shows recognition accuracies and *d*-prime mean values for Preferred and Non-preferred chords. Note that accuracies and *d*-prime values are significantly higher for Preferred chords. Error bars represent standard errors

Participants performed four runs of a two-phase *memorisation task*, composed by a first *memorisation phase* and a subsequent *recognition phase* (Fig. 1a). Procedures were identical in the four runs except for the set of musical chords presented in the memorisation phase which differed in each run. The chords presented in the memorisation phase in each run are reported in Table 1. The order of presented chords in each run was randomized across participants, in such a way that each participant was presented with a different chord sequence. Each chord was presented in two out of four runs of the memorisation phase. Participants performed the four runs in a randomized order. Runs were numbered from 1 to 4 in Table1 for clarity of display reasons only. In the memorisation phase, each of the four selected chords (two major and two diminished chords out of the total set of eight triads) was presented three times in a random order. Following each chord, a central fixation-cross remained on screen for 0.5 s, then a small stylized note on screen signaled the participants to sing out loud the previously presented chord for 5 s. We asked participants to actively repeat the previously heard chord to verify that they were actually paying attention, and that they all employed the same memorisation strategy (repetition out loud has been shown to be a very effective memorisation strategy by previous research; Lafleur & Boucher, 2015). Subsequently, a question mark appeared at the centre of the screen indicating to participants they had to evaluate the beauty of the previously presented musical chord (*Aesthetic Judgement*) on a 1 to 9 Likert scale (1 indicates ugly triads and 9 beautiful triads; Sarasso, Ronga, et al., 2019b; Sarasso, Ronga, et al., 2020b). The question mark remained on the screen for 5 s, followed by the next trial after a 1 s ITI.

At the end of the memorisation phase, participants performed a *distraction task* (duration 5 minutes), where they had to detect luminous targets flashed on a black screen (Ronga et al., 2018; Sarasso, Ninghetto, et al., 2019a). Following the distraction task, participants underwent to the *recognition phase*. Eight chords were played one by one (arpeggiated triad chord duration 2.5 s). Notably, only 50% of chords had been presented before in the previous memorisation phase. Following each chord presentation, participants were asked to look at the fixation cross for 1 s and then respond whether they had already heard the chord in the previous phase or not by pressing two adjacent keys on the keyboard. Subjects’ responses were collected and automatically recorded by E-Prime (Psychology Software Tools, Inc. USA). Following subjects’ response, a 2-s break preceded the beginning of the next trial.

#### Data analysis

Each triad chord (MAJ 1, MAJ 2, MAJ 3, MAJ 4, DIM 1, DIM 2, DIM 3, DIM 4) was presented once in each run of the recognition phase, for a total of four collected responses per chord in the whole experiment (four runs). Response ACC—that is, subjects correctly reporting whether they already heard the chord or not—were averaged across chord type (major vs. diminished). As a result, for each participant, we obtained a mean ACC value ranging from 0 to 4 for each chord type. Aesthetic judgements (AJs) collected during the memorisation phase were also averaged across chord types to obtain one mean AJ value per chord type per participant.

To exclude that possible ACC modulations were due to a response bias and to further explore subjects’ ability to recognize previously heard chords, we calculated *d-*prime values (i.e. *z*-transformed hit rates minus *z*-transformed false alarm rates; Rotello, 2017). Mean single subjects’ ACC, AJs, and *d*-prime values served as input for the subsequent analyses. We performed a two-tailed paired-samples *t* tests on (1) mean AJs and (2) mean ACC for MAJ and DIM chords.

#### Analyses based on aesthetic preference judgements

To explore the relation between subjective preference and accuracy, we split ACC and *d*-prime values into two groups (preferred and non-preferred) according to individual AJs, so that for each participant the ACC and the *d*-prime values corresponding to the preferred chord type (the one with greater mean AJ) were assigned to the preferred group, whereas the ACC and the *d*-prime values corresponding to the non-preferred chord type (the one with smaller mean AJ) were assigned to the non-preferred group. A two-tails paired-samples *t* test was performed on preferred and non-preferred ACC and *d*-prime values.

## Results

As expected, MAJ chords were significantly more appreciated than DIM chords (average ± *SD*, MAJ = 4.927 ± 0.984; DIM = 4.52 ± 0.923), *t*(59) = 5.66, *p* < .001, 95% CI [0.264, 0.552], Cohen’s *d* = 0.42663 (see Fig.1b), thus replicating the results of previous studies on aesthetic appreciation and consonance (Bowling et al., 2017; Sarasso, Ronga, et al., 2019b). Fourteen participants (23%), however, preferred mildly dissonant DIM chords. Interestingly, memorisation ACC corresponding to MAJ versus*.* DIM chords were not significantly different, *t*(59) = 0.92, *p* = .359, 95% CI [−0.102, 0.277]). Crucially, however, ACC of the preferred chords (see Methods section, Experiment 1) were significantly higher than ACC of the non-preferred ones (Preferred = 2.263 ± 0.486; Non-preferred = 2.05 ± 0.484), *t*(59) = 2.33, *p* = .023, 95% CI [0.03, 0.395], Cohen’s *d* = 0.3 (percentages in Fig. 1b). That is, ACCs differed only once we grouped them according to individual subjective preferences, with higher memorisation performances for more appreciated chords, independently from the chord type. Furthermore, this result was confirmed by the *t* test comparing *d*-prime values corresponding to preferred versus non-preferred chords (Preferred = 0.834 ± 1.574; Non-preferred = −0.007 ± 1.18), *t*(59) = 3.11, *p* = .003, 95% CI [0.3, 1.381], Cohen’s *d* = 0.402 (see Fig. 1b). All significant results survived Benjamini–Hochberg correction for multiple comparisons (false discovery rate = 10%; total number of tests in the study = 4).

## Experiment 2: Mismatch detection task (EEG)

### Methods

#### Participants

Twenty-two right-handed healthy volunteers participated in the study (14 females; age: 23.31 ± 1.76 years; scholarity: 15.26 ± 1.89 years). Sample size matched that of a previous EEG study by our group assessing the correlation between AJs and attention-related components of the auditory ERP in response to more and less consonant two-note just intervals (Sarasso, Ronga, et al., 2019b).

We excluded volunteers with a formal musical training, as well as professional players and singers. None of our participants played a musical instrument more than once in their life. All participants gave their written informed consent to participate in the study. The study conformed to the standards required by the Declaration of Helsinki and was approved by the local ethics committee (University of Turin) protocol number 121724.

#### Stimuli and apparatus

Standard and deviant stimuli consisted of musical just intervals with different frequency (Hz). Differently from Experiment 1, where the single notes composing the interval were presented in sequence, to explore the EEG response to each stimulus, we opted for the simultaneous presentation of the interval. Mismatch detection responses registered with the EEG are sensitive to the absolute pitch difference between frequency standard and frequency deviant sounds, with greater mismatch responses to more distant (in terms of pitch) deviant sounds (Näätänen et al., 2007; Sams et al., 1985). Given this evidence, and similarly to previous studies (Sarasso, Ronga, et al., 2019b), we employed fifth and tritones two-note intervals, instead of the three-note triads employed in Experiment 1. This allowed us to limit the difference in single notes frequency (Hz) between frequency deviant and frequency standard intervals (see Table 2) and, most importantly, to match such frequency difference across interval types. This frequency matching was necessary to highlight possible modulations driven by subjective AJs.

**Table 2:** Stimuli

Frequency of interval presentation	Frequency of note1 (Hz)	Frequency of note2 (Hz)	
		Fifth	Tritone
12.5% (Deviant trial)	200	133.33	142.22
75% (Standard trial)	230	153.33	163.55
12.5% (Deviant trial)	260	173.33	184.88

*Note.* Note1 and note2 were always played simultaneously for 50 ms. Three intervals with varying frequencies were displayed for each interval type. Each interval type had a constant frequency ratio between note1 and note2. For each interval type, intervals with note1 set at 230 Hz were considered Standard and were displayed in 75% of the trials. The remaining two intervals with note1 set at 200 and 260 Hz were considered Deviant and were displayed in 12.5% of the trials.

In Experiment 2, we employed fifth and tritone intervals, which, despite being far in terms of consonance, are composed by single tones which are very similar in terms of frequency (Hz). This was an essential feature, since EEG fluctuations are very sensible to changes in frequency (Sarasso, Ronga, et al., 2019b).

As in Experiment 1, intervals were tuned according to just intonation. Any interval tuned in this way is called a just interval. We employed two interval ratio types: fifths (more consonant) and tritones (less consonant). We created three different intervals for each of the two interval ratio types, by varying the frequency of the two notes while maintaining equal the ratio between notes frequency (see Table 2). In Table 2 we report the frequency of the two notes (note1 and note2) composing the six intervals employed in Experiment 2. Intervals were played via headphones for 50 ms. For both interval types, intervals with the frequency of note1 set at 230 Hz were presented 30 times per block and corresponded to Standard trials. The remaining two intervals (with note1 set at 200 or 260 Hz) were displayed only 5 times per block and corresponded to Deviant trials.

Participants sat at a table with their eyes open, facing a 53 cm (diagonal) computer screen. The screen centre was aligned with their trunk midline. Participants’ arms were resting on the corresponding leg.

#### Experimental procedures

**Fig. 2:**
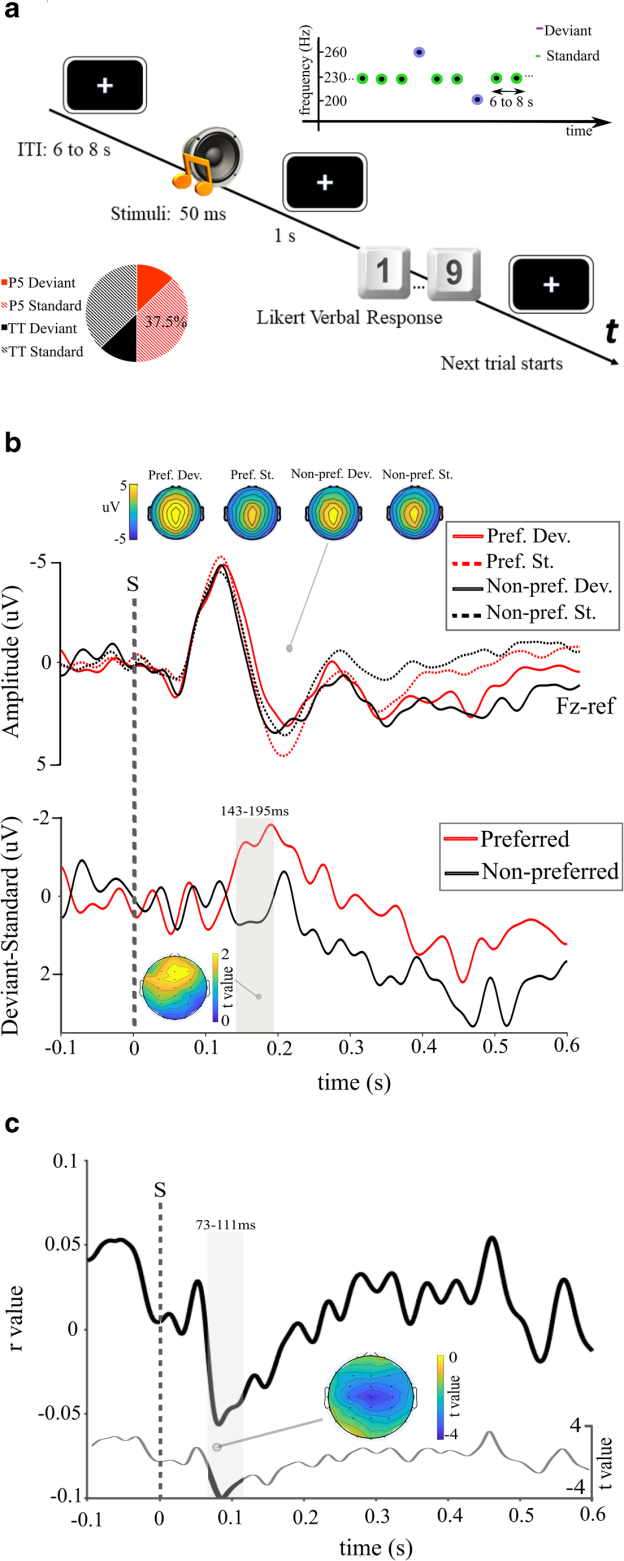
**a. Experimental procedures.** The trial timeline for the AJ task: after the interval was played participants remained still for one second and then they gave their answer. The pie chart represents the percentage of deviant (25%) and standard (75%) intervals for both fifth intervals and tritones. The graph at the top represents an example train of nine intervals, with two Deviant and seven Standard intervals. The *y*-axis represents the frequency of note one of intervals (see Methods, Experiment 2). **b. Mismatch detection responses.** Top section: Grand-average ERP for different standard and deviant interval types. Scalpmaps represent signal amplitudes registered at 200-ms post-onset across experimental conditions. Bottom section: Average mismatch negativity (MMN) waveforms for fith intervals and tritones (difference between ERP of deviant and standard intervals registered on Fz). Shaded areas respresent significant time-clusters higlighted by the point-by-point t-test. The scalpmap represents *t* values across channels at 160 ms post-onset. **c. trial-by-trial correlation.** Results from the trial-by-trial point-by-point correlation analysis. The solid black line represents mean *r* values at channel Cz obtained by correlating single trials amplitudes with the corresponding AJ. The thin grey line represents *t* values from the point-by-point *t* test comparing single subjects’ *r* values against 0 at channel Cz. The shaded area represents the significant cluster evidenced by the analysis at channel Cz, corresponding to the N1 component of the ERP. The scalpmap in Panel **c** shows *t* values from the point-by-point *t* test across channels at 100 ms post-onset. P5 = Fifth intervals; TT = tritone intervals; S = Stimulus onset

Participants performed one single run of the AJ task for each interval type (Fifth, Tritone). Differently from previous studies (Crespo-Bojorque et al., 2018) dissonant and consonant chords were never intermixed in the same experimental run. Instead, we evaluated the presence of a different deviancy effect among different consonant intervals as compared with different dissonant intervals. Each run was composed of two identical blocks. In each block, participants evaluated the beauty of forty presented intervals using a Likert scale ranging from 1 to 9 (where 1 corresponded to “The ugliest chord I can imagine” and 9 corresponded to “The most beautiful chord I can imagine”). Eighty intervals (60 [75%] Standard; 20 [25%] Deviant) were displayed in each run for a total of 160 stimuli in the whole experiment. The trial timeline is depicted in Fig. 2. Intervals were presented in a random order (with different trial sequences for each subject) after a variable intertrial interval, ranging from 6 to 8 s. Note that the intertrial interval was longer than in classical EEG oddball paradigms to allow the collection of a trial-by-trial AJ. Participants fixated a central white cross for the whole experiment. When they heard an interval, they were asked to wait (1 s) until the cross changed into a question mark and then verbally report their answer. AJs were recorded by the experimenter and were automatically registered (E-Prime 2.0 software, Psychology Software Tools, Inc., USA). Participants took a 5-minute break between the two runs. Each run lasted approximately 10 minutes.

#### Electrophysiological recordings and preprocessing

EEG was collected during the whole experiment with 32 Ag-AgCl electrodes placed on the scalp according to the extended International 10–20 system and referenced to the nose. Electrode impedances were kept below 5 kΩ. The electro-oculogram (EOG) was recorded from two surface electrodes placed over the right lower eyelid and lateral to the outer canthus of the right eye. EEG activity was recorded with a HandyEGG (Micromed, Treviso, IT) amplifier and continuously digitized by at a sampling rate of 1024 Hz. Off-line EEG preprocessing and analyses were conducted with Letswave6 toolbox (Nocions, Ucl., BE) for MATLAB (The MathWorks, Inc., USA). Data were divided into epochs of 1.5 s, including 500 ms prestimulus and 1 s poststimulus intervals. Epochs were band-pass filtered (0.5–40 Hz; Garrido et al., 2008; Y. Zhang et al., 2018) using a fast Fourier transform filter. Filtered epoched data were baseline corrected using the interval from −0.5 to 0 s as a baseline. Ocular artefacts were eliminated using Independent Component Analysis (ICA; Jung, Makeig, Humphries, Lee, McKeown, Iragui & Sejnowski, 2000). ERPs belonging to the same interval type and to the same condition (standard vs. deviant) were then averaged, to obtain four average waveforms (i.e. Fifth Standard, Fifth Deviant, Tritone Standard, Tritone Deviant) for each subject.

#### Data analysis

AJs from the AJ task collected in Experiment 2 were averaged across interval types to obtain two average values per participant, one for fifth intervals and one for tritones. Single subjects’ averaged AJs were entered in a two-tailed t-test comparing AJs for fifth intervals and tritones at a group level.

For each participant and for each interval type separately, mismatch detection responses were obtained by subtracting the ERP elicited by standard intervals from that elicited by deviant intervals (Näätänen et al., 2007). This passage was crucial to verify the presence of mismatch detection responses (i.e. larger responses for deviant vs. standard stimuli) even in our modified version of the oddball task. Subsequently, as in Experiment 1, for each participant we assigned average mismatch responses corresponding to fifth and tritone intervals to the preferred and non-preferred group, based on AJs collected during the AJ task. For each participant, average mismatch responses (Deviant–Standard) corresponding to the preferred interval (the one with greater mean AJ) were assigned to the preferred group, whereas average mismatch responses corresponding to the non-preferred interval (the one with smaller mean AJ) were assigned to the non-preferred group. We then performed a point-by-point *t* test (Harris et al., 2018; Novembre et al., 2018; Ronga et al., 2013; Valentini et al., 2014) with cluster-based permutation correction for multiple comparisons (1,000 permutations; alpha level = 0.05; percentile of mean cluster sum = 95; minimum number of adjacent channels = 2) on differential mismatch detection responses (Deviant–Standard). The test compared single subjects’ mismatch detection response amplitudes for preferred versus non-preferred intervals at each time point, for each channel separately.

Furthermore, to attempt replicating previous results evidencing a correlation between attention related components such as the N1/P2 complex of the ERP and trial-by-trial fluctuations in subjective AJs, we performed the following correlation analysis: For each single participant, we computed a point-by-point trial-by-trial correlation (Novembre et al., 2018; Sarasso,  Ronga, et al., 2020b; Sarasso, Ronga, et al., 2019b) between the amplitude of the EEG responses from single trials (*N* = 80) registered during the AJ task and the corresponding AJ (see Experimental Procedures). The outcome of the correlation analysis was a 1.5 s (from 0.5 s pre-onset to 1s post-onset) long time series of *r* values for each channel for each subject. This constituted the input for a group-level two-tailed point-by-point *t* test with permutation-based correction for multiple comparisons (1,000 permutations; alpha level = 0.05; percentile of mean cluster sum = 95; minimum number of adjacent channels = 2). The test compared single subjects’ correlation coefficients against the constant 0 at each time point. This allowed to identify time-clusters containing signal amplitudes which significantly correlated with AJs.

### Results

Two participants were excluded from subsequent analyses due to technical problems while recording their EEG. The remaining 20 participants were included in the subsequent analyses. AJs collected in Experiment 2 replicated previous findings (Bowling et al., 2017): More consonant intervals were on average more appreciated (fifths = 4.01 ± 1.25) than more dissonant intervals (tritones = 3 ± 1.22). Preferences were very stable: On average, all 20 participants rated fifth intervals as more beautiful than tritones. At a group level, AJs for the two interval types were significantly different (*t* = 5.99, *p* < .001, 95% difference CI [0.65, 1.36]). Therefore, interval types and preferred/non-preferred intervals coincided in Experiment 2. Accordingly, all single participants’ mismatch responses corresponding to fifth and tritone intervals were assigned respectively to the preferred and non-preferred groups in subsequent analyses.

Results from the trial-by-trial correlation analysis evidenced a significant cluster at Cz (73–111 ms; see Fig. 2c) corresponding to the N1 component, peaking over Cz at 105-ms post-onset. This result indicates that subjective trial-by-trial AJs significantly correlate with the amplitude of the attention-related component N1, with larger N1 amplitudes associated with more appreciated stimuli at the single trial level.

ERPs from deviant and standard intervals registered from F_z_ are reported in Fig. 2b. Grand-average waveforms were comparable with those reported in previous studies on auditory frequency processing (Sams et al., 1985). For both preferred and non-preferred interval types, mismatch detection responses (Deviant–Standard difference waveforms) showed a negative peak at approximately 200 ms post-onset, in accordance with previous findings (Sams et al., 1985). The point-by-point *t* test performed on mismatch detection responses (preferred vs. non-preferred) registered on F_z_ revealed one single significant time cluster centred on the average waveform negative peak (143–195 ms; see Fig. 2b). As expected, mismatch detection responses were significantly larger for more appreciated consonant intervals. Results were comparable among fronto-central electrodes. We therefore show only results from the *t* test performed on F_z_ where differences in the mismatch detection performances are more pronounced.

## Discussion

In this study, we demonstrate the existence of a relationship between lower and higher-order learning phenomena and aesthetic appreciation, as indicated by (1) better memorisation performances (accuracy rate and *d*-prime values) for subjectively preferred as compared with non-preferred triad chords (see Fig. 1b); (2) the trial-by-trial correlation between amplitude fluctuations of the N1 attention related component and subjective AJs; and (3) enhanced electrophysiological mismatch detection responses, evidencing ameliorated implicit learning of sensory regularities for preferred intervals (see Fig. 2). Moreover, it is important to notice that, in Experiment 1, chord type per se (consonant vs. dissonant) did not influence memorisation performances. This result is coherent with those of previous studies investigating short-term memory for just-tuned consonant and dissonant dyad intervals, which demonstrated that small-integer ratio dyads (consonant intervals) showed no innate memory advantage; musicians’ and non-musicians’ recognition of consonant intervals was no better or worse than that of dissonant intervals (Rogers & Levitin, 2007). As we will discuss below, these results, together with our findings, seem to support the hypothesis that memory advantages are independent from consonance per se, while memory performances might be directly linked to subjective preferences.

Overall, the present findings, indicating enhanced memorisation performances for subjectively preferred intervals and chords, may be considered as supporting evidence to our hypothesis of a correlation between perceptual learning and subjective aesthetic appreciation. In previous research we showed that more appreciated intervals boost perceptual processing, inducing an automatic re-orienting of attentional resources towards the sensory inputs (Sarasso, Neppi-Modona, et al., 2020a). This effect, also evident in Experiment 2, is reflected in the significant enhancement of attention-related electrophysiological responses (Sarasso, Ronga, et al., 2020b; Sarasso, Ronga, et al., 2019b) and in the consequent improvement of perceptual performances for more appreciated stimuli (Sarasso, Ronga, et al., 2020b; Spehar, Wong, van de Klundert, Lui, Clifford & Taylor, 2015). We propose that a similar mechanism might underlie the behavioural results of Experiment 1. Our interpretative hypothesis is that preferred intervals elicited increased sensory activations and improved perceptual implicit learning in the memorisation phase via an automatic attentional modulation, which in turn triggered enhanced memorisation performances in the recognition phase. In other words, the results of Experiment 1 seem to indicate that the previously demonstrated beauty-related boost in low-level perceptual processing might also induce a learning gain at higher levels. However, to the best of our knowledge, evidence directly exploring the beauty-driven modulation of low-level perceptual learning phenomena is still missing. With the final aim of verifying the presence of such a mechanism at an implicit level, we performed Experiment 2.

Results of Experiment 2 are twofold. First, our findings confirm previous studies evidencing a correlation between AJs and early attentional electrophysiological responses to more and less consonant musical intervals (Sarasso, Ronga, et al., 2019b) and images with more or less natural frequencies content (Sarasso, Ronga, et al., 2020b). The N1 component amplitude has been frequently described as an index of attentional engagement (Alho, 1992; Fritz et al., 2007; Giuliano et al., 2014; Wilkinson & Lee, 1972). Indeed, it has been shown that valid spatial and temporal cues can enhance the auditory N1 component (Hillyard & Anllo-Vento, 1998; Hötting et al., 2003). Fluctuations in the auditory N1 component are also modulated by task-relevance, stimulus saliency, and predictability (Lange, 2013; Zani & Proverbio, 2012). In accordance with previous findings (Regnault et al., 2001; Virtala et al., 2014), trial by-trial fluctuations in N1 voltages registered during Experiment 2 significantly correlated with single trial AJs (see Fig. 2). Moreover, as we expected, mismatch detection responses (i.e. responses to deviant intervals minus responses to standard intervals) were significantly more pronounced for more appreciated interval types. The increase in mismatch detection responses is usually interpreted as a correlate of optimal implicit statistical learning of sensory regularities (Garrido et al., 2016; Näätänen et al., 2007) and is impaired in a number of pathological conditions (Garrido et al., 2009) and learning impairments (Cantiani et al., 2019). Interestingly, the enhancement of mismatch detection has been demonstrated to correlate also with higher-order learning phenomena, such as the acquisition of new linguistic skills, thus indicating that improved low-level perceptual learning mechanisms might predict higher-order learning outcomes (Winkler et al., 2003; Ylinen et al., 2010).

Overall our behavioural and electrophysiological results, in accordance with previous evidence, show that subjective aesthetic appreciation is related to an automatic re-orienting of attention toward the sensory stimulation, leading in turn to the enhacement of lower-level (i.e. mismatch detection) and higher-level (i.e. memorisation) learning. What might explain such attentional capture and increased implicit perceptual learning for more appreciated intervals?

Previous neurocomputational theories suggested that, in order to maximize epistemic value, intelligent systems (biological and artificial) have developed an intrinsic feedback on information gains (Gottlieb et al., 2013). According to this view, the brain automatically generates intrinsic rewards in response to stimuli with high informational content, signaling to the nervous system to focus on present sensory stimulation to learn something new. As we previously discussed, higher AJs seem to be assigned to stimuli valued as more profitable in terms of informational content (Biederman & Vessel, 2006; Chetverikov & Kristjánsson, 2016; Consoli, 2015; Perlovsky, 2014; Perlovsky & Schoeller, 2019; Schmidhuber, 2009). In other words, aesthetic appreciation may emerge anytime the cognitive system senses a refinement of the mental representations of the environment (Muth & Carbon, 2013; Schoeller & Perlovsky, 2016; Van de Cruys & Wagemans, 2011). Accordingly, the perception of beauty may be considered as a feedback allowing the individual to discriminate between informationally profitable (i.e. leading to learning progresses) and noisy (i.e. “unlearnable”) signals. This might explain the overall preference for more consonant intervals, given the evidence that consonant intervals are processed more fluently than dissonant intervals (Crespo-Bojorque et al., 2018; Crespo-Bojorque & Toro, 2016; Masataka & Perlovsky, 2013). Crespo-Bojorque et al. (2018) found that dissonant infrequent intervals played within a stream of frequent consonant intervals elicited larger mismatch negativities (MMN) as compared with the opposite condition (i.e. infrequent consonant intervals embedded within a dissonant context). The authors interpret their results as evidence for an early processing advantage for consonant over dissonant intervals. Although it is impossible to exclude that these results were also driven by the easier detection of dissonant sounds within a consonant context, which more closely resembles everyday musical experience, the interpretation suggested by the authors confirms the present findings. Indeed, since electrophysiological mismatch detection responses reflect the extent to which sensory information is weighted according to its estimated reliability (also referred as precision-weighted prediction errors; Quiroga-Martinez et al. 2019), it might be argued that in both our and Crespo-Bojorque’s study, mismatch detection responses elicited in a consonant context were enhanced by the automatic up-weighting of consonant sensory inputs. Apparently, a more consonant sensory context, similarly to a low-entropy sensory context, induces the brain to estimate the inputs as more reliable (Quiroga-Martinez et al., 2019). It has also been suggested that our auditory cortices are generally more tuned to process consonant sounds (Bowling & Purves, 2015; Bowling et al., 2017) due to the similarity with human vocalizations (Crespo-Bojorque & Toro, 2016; Toro & Crespo-Bojorque, 2017). However, personal experiences, as musical training and listening, seem to be able to modulate these general trends (Crespo-Bojorque et al., 2018). Accordingly, AJs, processing advantages and implicit perceptual learning do not always correlate with consonance, but can vary according to some contextual (Brattico et al., 2013; Mencke et al., 2019; Pelowski et al., 2017), experiential (Koelsch et al., 2019), cultural (Lahdelma & Eerola, 2020; McDermott et al., 2016), and personal factors (Brattico et al., 2009; McDermott et al., 2010; Plantinga & Trehub, 2014; Proverbio et al., 2016). Professional musicians, as an example, show larger MMNs (Crespo-Bojorque et al., 2018), a superior automatic discrimination (Brattico et al., 2009), and higher aesthetic appreciations (Istók et al., 2009; Müller et al., 2010; Schön et al., 2005; Smith & Melara, 1990) of non-prototypical dissonant intervals. However, the evidence for the hypothesis that musical expertise facilitates neural processing of dissonant musical stimuli is still conflicting. As an example, Linnavalli et al. (2020) found that dissonant deviant chords (embedded within a dissonant context) elicited similar MMN responses for musicians and non-musicians and hypothesize that the facilitating effects of musical expertise might emerge in higher stages of auditory processing, influencing only behavioural discrimination.

Altogether, cultural familiarity, individual experiences and even personality traits may induce the nervous system to reinterpret some specific sensory signals usually valued as “noisy” as more informationally profitable (Hsu et al., 2015; Mencke et al., 2019). As an example, beside purely acoustic factors, tritones might be usually disliked because of Western music aesthetic conventions (Partch, 1974). This effect is crucial in showing that the weighting of the sensory input, rather than being *aprioristically* defined, is sensitive to contextual variability (such as frequency of exposition, contextual relevance) and may differ across individuals and even within the same individual, from time to time (Ronga et al., 2017; Van Beers et al., 2002). This might explain the differences in subjective AJs and memorisation performances across triad types in Experiment 1. Coherently with this idea, in Experiment 2, results from the trial-by-trial correlation strongly suggest a direct relation between subjective aesthetic appreciation and the hypothesized attentional up-weighting of auditory inputs. Indeed trial-by-trial fluctuations in the amplitude of attentional N1 component correlate with single trials AJs independently from interval type.

As a limit of Experiment 2, we must point out that the result on mismatch responses to preferred versus non-preferred intervals, although in line with our hypothesis of a correlation between perceptual learning and subjective aesthetic appreciation, does not exclude that enhanced implicit learning of sensory regularities is exclusively related to interval consonance, rather than specifically to subjective AJs. Contrarily to Experiment 1, where we employed more similar (major and diminished) chords in terms of consonance/dissonance, in the sample of participants included in Experiment 2, individual preferences did not vary across more and less consonant (fifth and tritone) intervals. In Experiment 2 preferences were all oriented toward more consonant fifth intervals, which renders it impossible to disjoint the effect of mere acoustic difference of stimuli from subjective preference. These results are coherent with previous studies showing a inverted-U shape for preferences: when dissonance is relatively low, preference does not decrease with increasing dissonance, while for relatively higher degrees of dissonance, preference decreases with increasing dissonance (Lahdelma & Eerola, 2016a). This might explain why some participants in Experiment 1 preferred mildly dissonant diminished chords. Still, results from Experiment 2 do not allow a clear-cut dissociation between consonance and likings, thereby limiting the evidence in favor of a selective correlation between perceptual learning and AJs. However, fifth and tritone intervals, despite being very far in terms of consonance, are composed by single tones which are very similar in terms of frequency (Hz). This was essential to exclude that EEG fluctuations were exclusively related to changes in frequency (Sarasso, Ronga, et al., 2019b). Further research, aiming to extend the comprehension of the relation between perceptual learning and AJs beyond our preliminary and methodologically constrained study, might employ intervals that reside on less extreme points of the consonance/dissonance continoum, which would likely induce greater variability in individual preferences. Furthermore, less culturally loaded stimuli would ideally lead to less polarized preferences (Lahdelma & Eerola, 2020).

In a follow-up study which is curretly under review (Sarasso, Neppi-Modona, et al., 2021a), we employed a roving paradigm to compare deviant and standard responses to fifth and tritone intervals. In our experimental sample some participants preferred fifth intervals over tritones, and we found that, similarly to Experiment 1, MMN were significantly different only when comparing subjectively preferred and non-preferred intervals, but not when comparing consonant (fifth) versus dissonant (tritone) intervals. This result further points to a significant correlation between implicit learning and subjective aesthetic preferences, independently from stimuli acoustic features.

As a further limitation of the present study, we did not collect any data regarding participants’ musical expertise or previous musical experience which, as we previously discussed, might mediate the influence on cognitive processes (Rogers & Levitin, 2007) and electrophysiological responses (Brattico et al., 2009; Crespo-Bojorque et al., 2018) triggered by more and less consonant sounds. Future studies should address this point by controlling for individual musical skills and listening expertise by means of ad hoc musical sophistication inventories, such as the OMSI (J. D. Zhang & Schubert, 2019) or the Gold-MSI (Müllensiefen et al., 2014) indexes. Moreover, in Experiment 1 we employed only two chord types. Future studies are needed to replicate our behavioural investigation of memorisation performances for musical stimuli employing a variety of chord types distributed across a wider spectrum of sensory consonance. Results from Experiment 1, indeed, might not be replicated employing more dissonant sonorities, which might create cognitive interference (see e.g. Masataka & Perlovsky, 2013). By this means, it would be possible to determine to what extent consonance level can overrule the effect of subjective preferences.

Altogether, our electrophysiological and behavioural results may be interpreted within a wider theoretical framework, suggesting that subjective aesthetic appreciation may represent the conscious feedback of optimal learning processes (Perlovsky & Schoeller, 2019; Schmidhuber, 2009; Schoeller & Perlovsky, 2016; Van de Cruys & Wagemans, 2011). Indeed, sequences with high information content (e.g. unexpected changes) in musical pieces have been shown to induce a state of higher arousal (Egermann et al., 2013) associated with aesthetic pleasure (Grewe et al., 2009). Hence, aesthetic pleasure could represent the intrinsic hedonic motivation to pursue further learning progresses and information gains (Biederman & Vessel, 2006; Sarasso, Neppi-Modona, et al., 2020a). From a philosophical perspective, it has been suggested that the intensity of the felt sensation elicited by a beautiful object (which, on a lower level of description, corresponds to an optimal learning process) represents the intrinsic reward further promoting a contemplative learning-oriented “aesthetic attitude” (Menninghaus, Wagner, Hanich, Wassiliwizky, Jacobsen & Koelsch, 2017; Menninghaus, Wagner, Wassiliwizky, Schindler, Hanich, Jacobsen & Koelsch, 2019). Interestingly, recent neuropsychological research is in accordance with the aesthetic philosophical literature in suggesting the existence of a tight connection between aesthetic appreciation and knowledge achievements. Within this framework, the investigation of subjective aesthetic appreciation appears crucial for the study of learning and memory. Future studies should assess whether the relationship between aesthetic appreciation and optimal learning processes can be fruitfully exploited to potentiate individual intrinsic motivation (Apter, 1983; Decoursey, 2016; Murayama, Matsumoto, Izuma & Matsumoto, 2010; Oudeyer, Gottlieb & Lopes, 2016) and memory retrieval (Lehmann & Seufert, 2018; Proverbio & De Benedetto, 2018) in activities such as teaching and cognitive rehabilitation, especially in presence of attentional/memorisation deficits.
